# Novel Endogenous, Insulin-Stimulated Akt2 Protein Interaction Partners in L6 Myoblasts

**DOI:** 10.1371/journal.pone.0140255

**Published:** 2015-10-14

**Authors:** Michael Caruso, Xiangmin Zhang, Danjun Ma, Zhao Yang, Yue Qi, Zhengping Yi

**Affiliations:** Department of Pharmaceutical Sciences, Eugene Applebaum College of Pharmacy/Health Sciences, Wayne State University, Detroit, MI, United States of America; Tohoku University, JAPAN

## Abstract

Insulin resistance and Type 2 diabetes are marked by an aberrant response in the insulin signaling network. The phosphoinositide-dependent serine/threonine kinase, Akt2, plays a key role in insulin signaling and glucose uptake, most notably within skeletal muscle. Protein-protein interaction regulates the functional consequence of Akt2 and in turn, Akt2’s role in glucose uptake. However, only few insulin-responsive Akt2 interaction partners have been identified in skeletal muscle cells. In the present work, rat L6 myoblasts, a widely used insulin sensitive skeletal muscle cell line, were used to examine endogenous, insulin-stimulated Akt2 protein interaction partners. Akt2 co-immunoprecipitation was coupled with 1D-SDS-PAGE and fractions were analyzed by HPLC-ESI-MS/MS to reveal Akt2 protein-protein interactions. The pull-down assay displayed specificity for the Akt2 isoform; Akt1 and Akt3 unique peptides were not detected. A total of 49 were detected with a significantly increased (47) or decreased (2) association with Akt2 following insulin administration (n = 4; p<0.05). Multiple pathways were identified for the novel Akt2 interaction partners, such as the EIF2 and ubiquitination pathways. These data suggest that multiple new endogenous proteins may associate with Akt2 under basal as well as insulin-stimulated conditions, providing further insight into the insulin signaling network. Data are available via ProteomeXchange with identifier PXD002557.

## Introduction

Insulin-stimulated glucose uptake and metabolism in target tissues is regulated through intracellular protein-protein interactions, as well as by protein post-translational modifications, notably phosphorylation [1–3]. Dysregulation of insulin signaling may lead to several debilitating disorders such as insulin resistance, metabolic syndrome, type 2 diabetes (T2D), cardiovascular disease, and/or cancer [4–6]. Two canonical insulin-stimulated signaling pathways have emerged: the phosphatidylinositide 3-kinase (PI3K) and the mitogen-activated protein kinase (MAPK) signaling pathways [7]. However, the PI3K insulin-stimulated pathway carries out the primary metabolic functions while MAPK regulates cell survival and mitogenesis [7]. The serine/threonine kinase, Akt, is a keystone mediator in the PI3K pathway, associating with numerous downstream proteins that affect metabolism, growth, and cell survival [8].

Akt, also known as protein kinase B (PKB), Rac-activated protein kinase (RAC-PK), or the cellular homolog of the transforming v-akt murine thymoma viral oncogene, exists in three isoforms—Akt1 (PKBα), Akt2 (PKBβ), Akt3 (PKBγ)—each encoded by a separate gene [9]. The three Akt isoforms share more than 80% amino acid sequence identity and contain major structural features such as an N-terminal pleckstrin homology (PH) domain that mediates lipid-protein and protein-protein interactions, a central kinase domain, and a hydrophobic C-terminal tail [10]. The akt1 isoform is the most predominately expressed across all tissue types, and homozygous knockout of Akt1 in mice display a reduced body weight phenotype [11]. Akt3 is predominantly expressed in nervous tissue [12], and homozygous knockout mice exhibit no aberrant decrease in body weight or glucose metabolism, but do display a reduction in brain mass [13]. Akt2 is primarily expressed in insulin-responsive tissues such as skeletal muscle and adipose [14].

Multiple studies have indicated that Akt2 is the primary isoform responsible for insulin-stimulated glucose uptake in humans as well as rodents and dysfunctional Akt2 is associated with insulin resistance and impaired glucose tolerance. Akt2 homozygous knockout mice (-/-) exhibit a severe diabetic phenotype, resulting in hyperglycemia, glucose intolerance, and hyperinsulinemia [15]. Additionally, calorie restricted Akt2 KO (-/-) mice exhibit impaired 2-deoxyglucose uptake despite elevated (compensatory) Akt1 activation in muscle [16]. In obese, insulin resistant Zucker rats, Akt2 expression is reduced by more than half, while Akt1 expression remains unaffected in muscle; however, both insulin-stimulated Akt1 and Akt2 activity is significantly diminished in muscle but not adipose [17]. This suggests that Akt’s involvement in insulin action maybe tissue- and isoform-specific in both expression and activation. Insulin-stimulated human muscle from obese, insulin resistant individuals display a decrease in Akt2 activity but not Akt1 compared to lean, healthy counterparts [18]. The expression of Akt2 and phosphorylation of Ser474 after a hyperglycemic episode in obese subjects was significantly decreased following *in vivo* insulin stimulation compared to after near-normoglycemic remission [19]. Knockdown (siRNA) of Akt2 in cultured myoblasts and myotubes derived from human rectus abdominus displayed decreased insulin-mediated glucose uptake, whereas Akt1 knockdown had no effect on glucose uptake [20]. An autosomal dominant missense mutation, R-H274, which affects the activation segment and catalytic loop of Akt2 has been indicated to result in severe insulin resistance and diabetes [21]. Although genetic mutations in the coding region of Akt2 resulting in insulin resistance are rare [22], the loss-of-function mutation (R-H274) signifies the importance of Akt2 in intermediate glucose metabolism. Collectively, these studies demonstrate a critical role of Akt2 in maintaining insulin sensitivity and glucose homeostatis in humans and rodents.

Intracellular protein-protein interaction is a known means of propagating a cell signaling event. Proteins may interact for several reasons, including but not limited to altering each other’s function (e.g., activation; inhibition), promoting degradation (e.g., ubiquitination), and/or increasing half-life/stability (e.g., binding proteins). Literature search, along with database analysis, indicates that that there are 55 intracellular proteins that have previously been shown to interact with Akt2 (S1 Table). Most of the proteins were identified by yeast-two hybrid and/or in non-muscle cell types, such as hepatocytes or HEK cells. Recently, Akt substrate 160 (AS160) was identified as an Akt interaction partner using a protein AGC phosphomotif (RXRXXpS/T) antibody and was subsequently characterized by mass spectrometry [23]. Akt (non-isoform specific Akt or pan-Akt) is known to interact with several kinases (e.g., protein kinase C) and phosphatases (e.g., PP2A) [24, 25], many of which play a role in mitogenesis, cell survival, growth, cell differentiation, and glucose uptake [7].

Proteomic approaches combining HPLC-ESI-MS/MS with co-immunoprecipitation (CO-IP), tryptic digest, and 1D-SDS-PAGE have been widely used to detect protein-protein interaction networks [26, 27]. Nonetheless, most of these studies were not conducted from bait proteins but instead used protein overexpression and/or epitope-tags, which can lead to false positive identification. Our system utilizes a label-free approach, without the use of protein overexpression or protein tags, to identify novel interaction partners and to quantify the changes in the abundance of endogenous proteins from multiple samples [28, 29]. Recently, we have improved this label-free approach, and discovered novel IRS1 interaction partners in skeletal muscle from lean healthy, obese non-diabetic, and type 2 diabetic patients, which has increased the number of partners identified and quantified across experimental groups [30].

As discussed above, Akt2 is the most abundant isoform of Akt in skeletal muscle cells that is critical in insulin signaling and glucose homeostasis in this cell type; nonetheless, only few insulin-responsive Akt2 interaction partners have been identified in skeletal muscle cells. Therefore, we hypothesize that there are novel Akt2 interaction partners in skeletal muscle cells, which are regulated in an insulin dependent manner. We used a label-free proteomics approach we developed for protein complexes to identify endogenous, insulin-stimulated Akt2 interaction partners in L6 myoblasts a widely used insulin-sensitive skeletal muscle cell model [31].

## Materials and Methods

**Fig 1:**
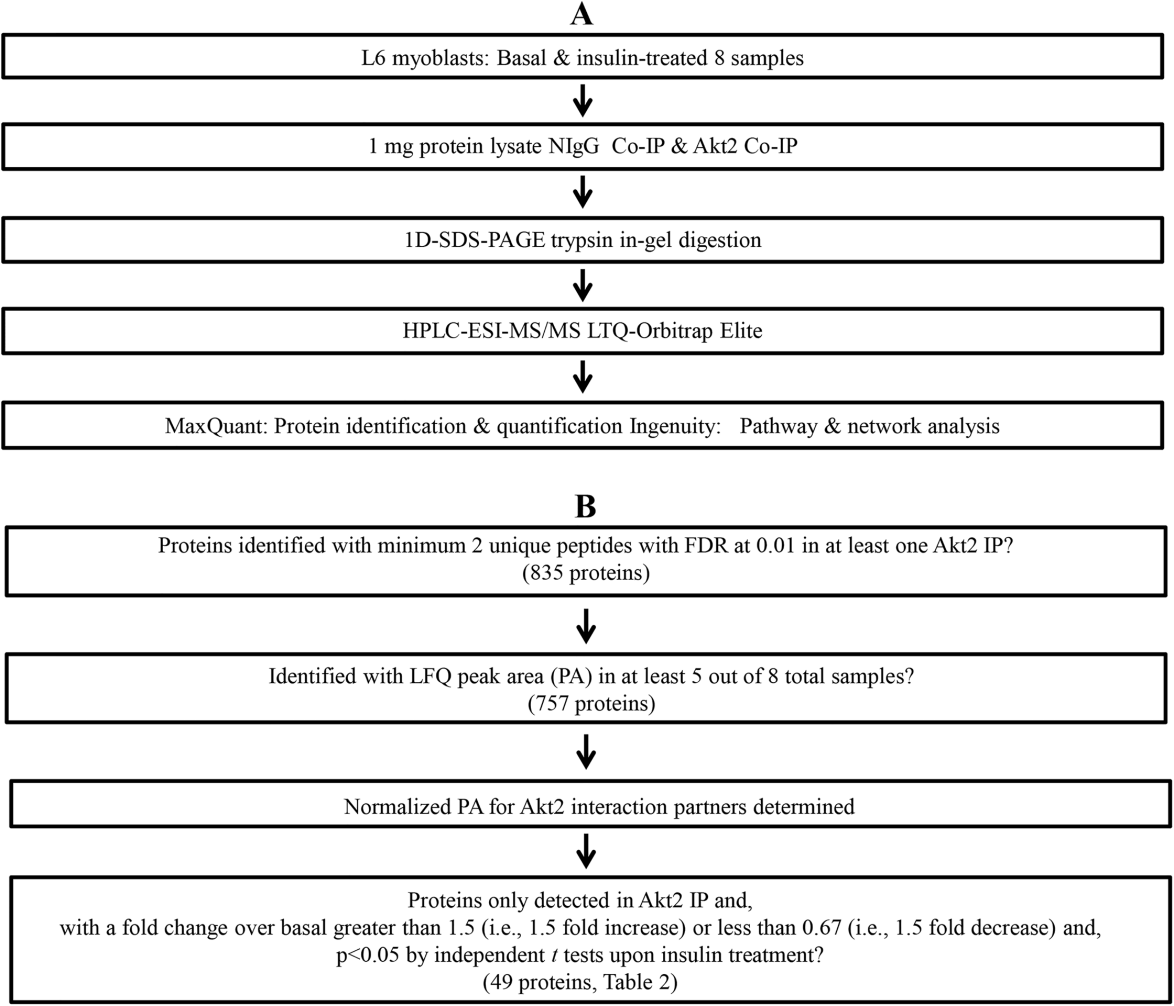
Experimental workflow design, and proteomics data acquisition and analysis. (A). Experimental workflow design. (B). Proteomics data acquisition and downstream analysis.

The outline of our approach is shown in Fig 1. The study started with L6 cell culturing, insulin treatment, cell lysis, and supernatant isolation; followed by immunoprecipitation of the “bait” protein (Akt2); followed by 1D-SDS-PAGE to resolve Akt2 co-interaction proteins; in-gel trypsin digestion to generate peptide fragments used in HPLC-ESI-MS/MS analysis for identification of co-immunoprecipitating proteins. The supernatant was pre-cleared by immunoprecipitation with non-specific antibodies (NIgG) to remove and identify non-specific proteins, which may contaminate the Atk2 Co-IP and minimizes false positives. Extensive bioinformatics and literature search were used to analyze proteomic data and to identify significantly enriched pathways, in which identified Akt2 interaction partners were involved.

### Antibodies and reagents

Anti-Akt2 for western blot was purchased from Cell Signaling Technologies, Beverly, MA (mouse; monoclonal; L79B2; 1:1000; a synthetic peptide surrounding Leu110 of human Akt2; RRID:AB_10544406). Anti-Akt2 (rabbit, polyclonal, 07–372, 2ug, peptide (C-RYDSLGSLELDQRTH) corresponding to amino acids 455–469 of human; RRID:AB_310562) and NIgG (rabbit; 12–370; 2ug; RRID:AB_145841), for immunoprecipitation, were purchased from EMD Millipore/Upstate, Billerica, MA. Anti-ROCK2 was purchased from Abcam, Cambridge, MA (mouse; monoclonal; ab56661; 1:500; Recombinant fragment corresponding to Human ROCK2 aa 1279–1388; RRID:AB_945286). DMEM medium was purchased from Invitrogen, Carlsbad, CA. Bradford reagent was purchased from Bio-Rad. Protein A sepharose beads and all other reagents were obtained from Sigma, St Louis, MO.

### Cell culture

The parental L6 myoblasts (CRL-1458) were purchased from ATCC, Manassas, VA and were maintained in DMEM medium supplemented with 10% fetal bovine serum and 5% penicillin-streptomycin-glutamine in a humidified atmosphere containing 5% CO_2_ and 95% air at 37°C. Cells were subcultured by trypsinization of subconfluent (<60%) cultures using 0.05% trypsin with EDTA. L6 myoblasts were seeded at a density of 8 x 10^5^ cells per 10 cm dish, and cultured until 100% confluent for two days. The cells were deprived of serum in DMEM medium supplemented with 0.2% BSA for 4 h at 37°C before treatment with insulin [28].

### Immunoprecipitation and Western Blot Analysis

Immunoprecipitation and western blot analysis are previously described in detail here [28]. Briefly, L6 myoblasts were incubated in culture medium with or without insulin (100 nM) for 15 min at 37°C. Cell culture medium was removed and cells were washed three times with PBS. One ml of lysis buffer (50 mM HEPES [pH 7.6], 150 mM NaCl, 20 mM sodium pyrophosphate, 10 mM NaF, 20 mM beta-glycerophosphate, 1% Triton, 1 mM Na_3_VO_4_, 1 mM phenylmethylsulfonyl fluoride, and 10 μg/ml leupeptin and aprotinin) was added to each plate; cells were scraped and collected in 1.5mL microcentrifuge tubes, and gently rotated for 15 min at 4°C to lyse the cells. The lysate supernatant was collected after brief centrifugation (10,000g for 20 min), while the pellet was discarded. Protein concentration of lysate was estimated by Bradford Assay (Bio-Rad) using bovine serum albumin (Sigma, St Louis, MO) as a standard. One mg of supernatant for each sample was precleared with 2 μg of NIgG primary antibody and 25ul of packed protein A agarose beads (Sigma, St Louis, MO) for 4hrs, used to detect non-specific binding. Protein A beads were pelleted and washed three times with 1 ml of lysis buffer. The supernatant was collected and incubated with 2 ug of primary antibody (Akt2) and 25ul of packed protein A beads overnight at 4°C with gentle rotation. Akt2 immunoprecipitates were then pelleted and washed three times with 1mL of lysis buffer. Samples were boiled in 15ul 2X Laemmli loading buffer with DTT (180 mM tris-HCl, 20% glycerol, 6% SDS, 125 mM DTT), pelleted, two times; and the eluate was collected and incubated with 6 ul of 0.1M iodoacetamide (IDA) (Sigma, St Louis, MO) for 45 minutes in the dark to reduce and alkylate cysteine residues, respectively. Samples were then resolved on 4–15% SDS-PAGE and proteins were visualized with Coomassie blue (Sigma Chemical Co., St. Louis, MO). All four basal and four insulin treated samples were harvested on the same day, which were paired and resolved on the same gel side by side to minimize gel to gel variations.

Alternatively, immunoprecipitate eluates were resolved on 4–15% SDS-PAGE, transferred onto nitrocellulose membranes, analyzed by western blotting with the appropriate primary antibodies (Akt2 and/or ROCK2) and HRP-linked secondary antibody, and the immune complex was detected by chemiluminescence.

### In-gel trypsin digestion, mass spectrometry, data analysis and bioinformatics

All were performed as previously described in reference [30]. Briefly, the gel lanes resulting from each experiment were cut into 4 slices for Akt2 and 3 slices for NIgG of approximately equal size. Please see S1 Fig for both NIgG and Akt2 gel. Each slice was cut into 1 mm cubes prior to digestion. The gel pieces were destained with 50% acetonitrile and 50% 40mM ammonium bicarbonate, and subjected to trypsin digestion at 37°C overnight. The resulting peptides were extracted and purified by solid-phase extraction (C18 ZipTip, Millipore, Billerica, MA), followed by high-performance liquid chromatography-electrospray ionization-tandem mass spectrometry (HPLC-ESI-MS/MS). HPLC-ESI-MS/MS was performed on a Thermo Finnigan LTQ-Orbitrap Elite fitted with a nanospray flex Ion source (Thermo Fisher, San Jose, CA). On-line HPLC was performed using an Easy-nanoLC II HPLC with a C18-reversed phase column (75 μm ID, 15 cm length) packed in-house with ReproSil-Pur C18-AQ μm resin (Dr. Maisch GmbH, Germany). Mobile phase, linear gradient of 2 to 10 ACN in 0.1% FA in 2 minute, followed by a step to 35% ACN in 75 minutes, and then a step to 43% ACN in 5 minute, followed by a step to 60% ACN in 2 minutes, and a step to 90% ACN in 2 minutes; flow rate, 200 nl/min.

A “top 20” data-dependent tandem mass spectrometry approach was utilized to identify peptides in the samples. In a top 20 scan protocol, a full scan spectrum (survey scan, 300–1650 Th) is acquired followed by collision-induced dissociation (CID) mass spectra of the 20 most abundant ions in the survey scan. The survey scan was acquired using the Orbitrap mass analyzer to obtain high mass accuracy and high mass resolution data (240,000 resolution), and up to 20 of the most intense ions were selected and subjected to fragmentation in the linear ion trap (LTQ). Dynamic exclusion was set at 30 seconds. The charge state rejection function was enabled with “unassigned” and “single” charge states rejected. By knowing the accurate mass and fragmentation pattern of the peptide, the peptide’s amino acid sequence can be reliably inferred.

Tandem mass spectra were extracted from Xcalibur ‘RAW’ files and searched against the forward and reversed Uniprot rat protein database (downloaded from www.uniprot.org on 3/6/2013) using Andromeda, the database search engine within the MaxQuant, one of the popular quantitative proteomics software packages [32–34]. The search variables used were: 6 ppm mass tolerance for precursor ion masses; 0.5 Da mass tolerance for product ion masses; digestion with trypsin; a maximum of two missed tryptic cleavages; variable modifications of oxidation of methionine, acetylation of protein N-terminus, and phosphorylation of serine, threonine, and tyrosine, as well as fixed modification of carbamidomethylation. Peptide false discovery rate (FDR) and protein FDR were both set at 0.01. Peak areas for each protein were obtained by selecting the Label-free quantification (LFQ) option in MaxQuant. Only proteins identified with minimum 2 unique peptides and with FDR at 0.01 were considered.

To be considered as an Akt2 interaction partner, a protein has to further satisfy following criteria: 1). Identified with LFQ peak area (PA) in at least 5 out of 8 total (basal and insulin-stimulated) Akt2 IP samples, and 757 proteins met this criterion; 2). Not detected with PA in the 8 NIgG control samples at all, and 296 proteins met this criterion; 3) with a significant change in their interaction with Akt2 upon insulin stimulation, and 59 proteins met this criterion.

To determine the relative quantities of Akt2 interaction partners in basal and insulin-treated conditions, the PA for each protein identified in a specific sample was normalized against the PA for Akt2 identified in the same sample, which results in Norm:*j*.

$$\mathrm{Norm}:j=\frac{\mathrm{PA}j}{{\mathrm{PA}\_}_{\mathrm{Akt}2}}$$

The normalization strategy is widely used in proteomics studies involving protein- protein interactions (26, 30), and uses the same concept used in Western blotting, in which the Western blot signal for an interaction protein is normalized against that for the protein serving as the “bait.” The normalized peak area for each Akt2 interaction partner, Norm:*j*, was compared between basal and insulin-treated conditions to assess effects of insulin on protein-protein interactions involving Akt2. Data are given in Table 1 as a representation of the normalization process as described above. The mass spectrometry proteomics data have been deposited to the ProteomeXchange Consortium via the PRIDE repository with the dataset identifier PXD002557.

**Table 1:** The effect of insulin on Akt2 interaction partner ROCK2. Peak area (PA) for each protein is normalized against Akt2 PA in the same sample.

**Basal**							
	**#1**	**#2**	**#3**	**#4**	**Mean**	**Stdev**	**SEM**
Total PA of a gel lane	1.10E+11	7.70E+10	6.72E+10	8.18E+10	8.41E+10	1.86E+10	9.29E+09
PA for AKT2	3.84E+09	3.70E+09	3.35E+09	1.50E+09	3.10E+09	1.08E+09	5.42E+08
PA for ROCK2	3.43E+05	0.00E+00	7.29E+05	0.00E+00	2.68E+05	3.47E+05	1.74E+05
Normalized peak area for ROCK2 normalized against AKT2	8.94E-05	0.00E+00	2.17E-04	0.00E+00	7.67E-05	1.03E-04	5.14E-05
**Insulin**							
Total PA of a gel lane	7.39E+10	6.48E+10	6.73E+10	7.48E+10	7.02E+10	4.91E+09	2.45E+09
PA for AKT2	1.74E+09	1.90E+09	2.28E+09	2.01E+09	1.98E+09	2.27E+08	1.14E+08
PA for ROCK2	7.60E+05	9.00E+05	9.84E+05	1.36E+06	1.00E+06	2.58E+05	1.29E+05
Normalized peak area for ROCK2 normalized against AKT2	4.37E-04	4.74E-04	4.32E-04	6.78E-04	5.05E-04	1.17E-04	5.83E-05

### Statistical analysis

Although a large number of proteins were assigned in at least one of 8 samples that were studied, a series of stringent filters were used to narrow the number of proteins that were used in comparisons among basal vs. insulin-stimulated, as described in [30]. This approach is diagrammed in Fig 1B. For comparisons to assess the effects of insulin stimulation, statistical significance was assessed using 2-tailed independent *t* tests, and differences were considered statistically significant at p<0.05.

### Bioinformatics analysis

Pathway analysis on Akt2 interaction partners were performed using Ingenuity Pathway Analysis (Ingenuity Systems, Inc., Redwood City, CA; www.ingenuity.com), which considers a pathway to be derived from a set of genes. IPA is widely used and contains biological and chemical/pharmacological interactions and functional annotations created by manual curation of the scientific literature [35–39].

To calculate statistical significance, IPA uses a hypergeometric distribution (Fisher's Exact Test), which calculates the probability (p) of finding a given number of genes (n) from the input data in each of the pathways [40]. Ingenuity queries a proprietary database of Canonical Pathways; a pathway was considered as significantly enriched if both the false discovery rate (FDR) [41] for the pathway was less than 0.01, and the pathway included at least 4 of the identified Akt2 partner genes.

## Results

From 4 independent biological comparisons (4 basal and 4 insulin stimulated samples, 4 slices/gel lane, 32 HPLC-ESI-MS/MS runs), a total of 835 unique proteins were identified in at least 1 out of the 8 Akt IP samples (Fig 1B). An additional 24 HPLC-ESI-MS/MS runs were performed for the corresponding NIgG samples under identical conditions to filter out non-specific protein binders.

**Fig 2:**
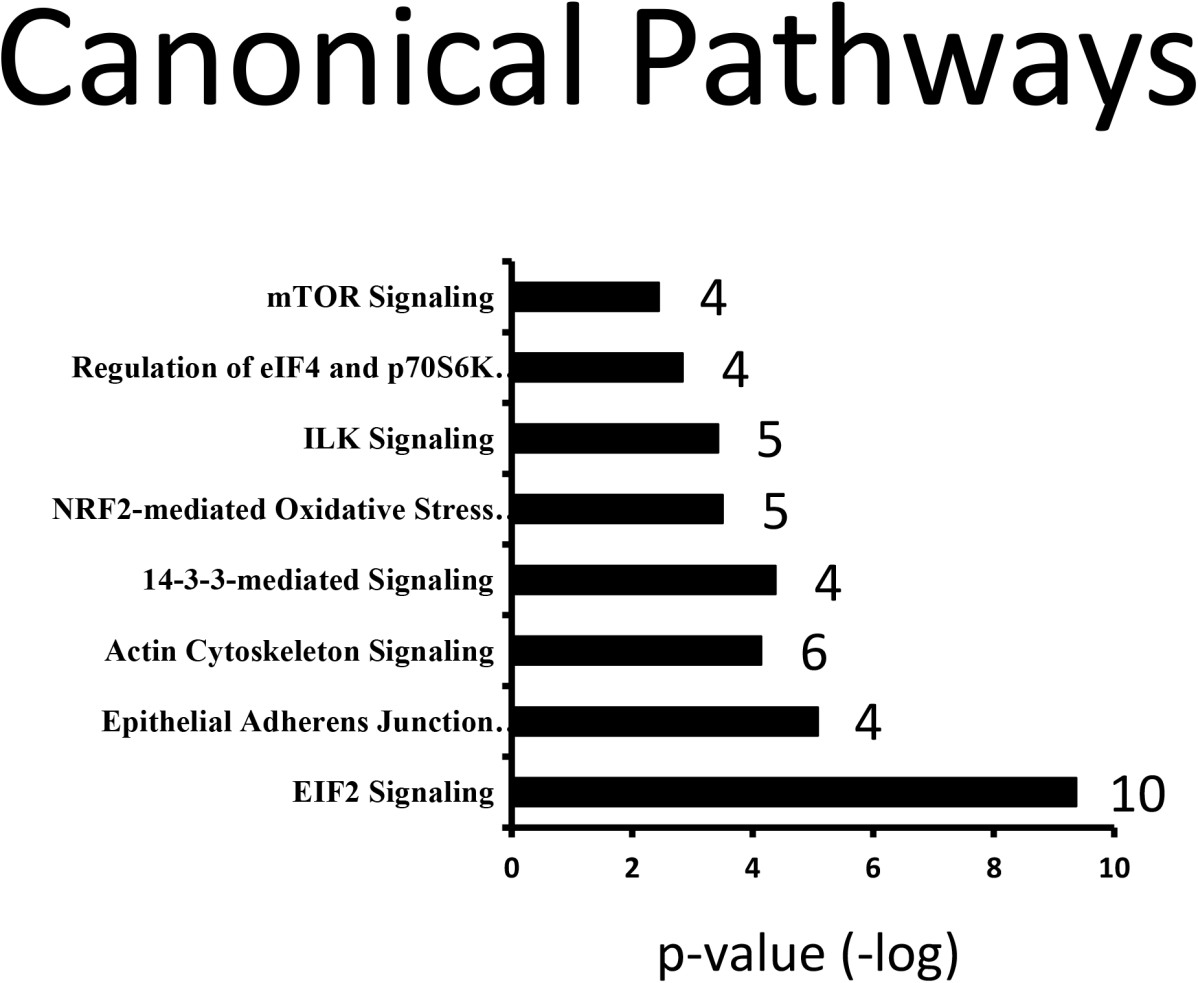
Significantly enriched canonical pathways for the 49 significant Akt2 interaction partners identified in this study. Pathway analysis was revealed by proteomics data and Ingenuity Pathways Analysis (IPA). The number of identified Akt2 interaction partners for a given pathway in this study are denoted besides each bar.

**Fig 3:**
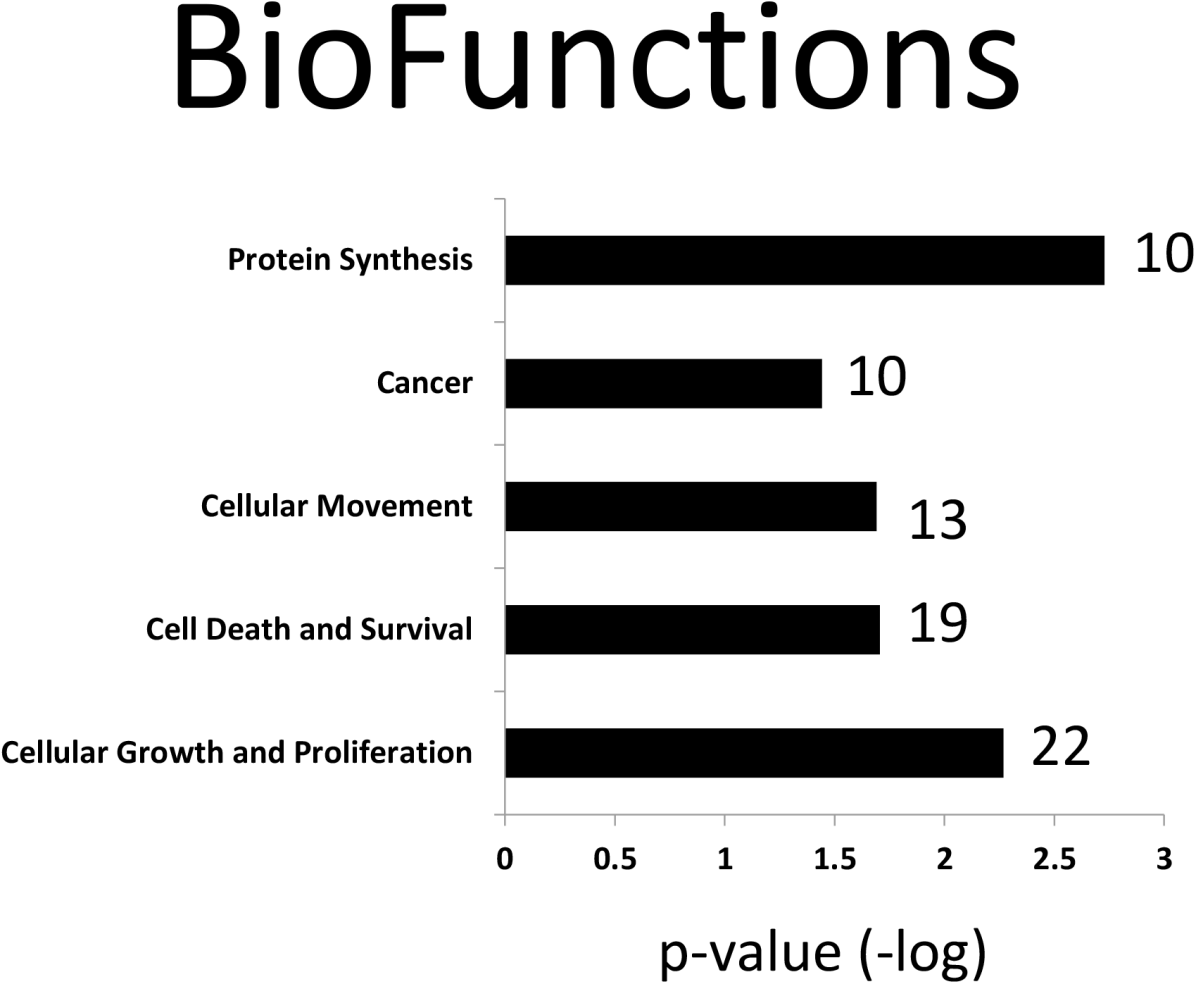
Significantly enriched biofunctions for the 49 Akt2 protein interaction partners in L6 myoblasts. The number of proteins identified for each biofunction is indicated in bold font, and the number of proteins for each biofunction with increased binding to Akt2 following the insulin treatment is indicated in the inset circle of each biofuntion, along with the –log(p-value) denoted inset the oval.

Akt2 was detected from all basal and insulin-stimulated cells but was not detected in any of the NIgG immunoprecipitates. Akt1 and Akt3 were not detected, suggesting the antibody we used is specific for Akt2. In total, 49 proteins met the criteria for classification as Akt2 interaction partners (Table **2**). Note that proteins may interact with Akt2 directly or indirectly, as a complex, through another protein that interacts with Akt2 directly. Among these 49 Akt2 interaction partners, 47 were previously unreported in any species. Elongation factor 1-alpha 1/2 and Tubulin have not been reported in skeletal tissue or muscle cells. Ingenuity Pathway Analysis (IPA) for the 49 Akt2 interaction partners identified in the study (plus Akt2 itself) indicated that 8 pathways were significantly enriched (p-value <0.01; including at least 4 identified Akt2 protein partners). The top-related canonical pathways are shown in Fig 2. Additionally, the top-related biofunctions are given in Fig 3. Note that biofunctions are manually grouped according to a common theme. These novel Akt2 interaction partners in L6 skeletal muscle cells may help understand the various roles that Akt2 plays in physiological and pathophysiological conditions in muscle.

**Table 2:** Significantly changed Akt2 protein interaction partners in L6 myoblasts upon insulin stimulation (n = 4, P<0.05). Each protein was detected in > 4 of the samples as well as not detected in any of the NIgG samples.

Gene Name	Protein Name	*Fold change over basal
Acadsb	Short/branched chain specific acyl-CoA dehydrogenase, mitochondrial	3.45±0.76
Akr1a1	Alcohol dehydrogenase [NADP(+)]	1.94±0.32
Cct6a	T-complex protein 1 subunit zeta	2.77±0.33
Cdv3	Protein CDV3 homolog	1.99±0.35
Cyfip1/2	Cytoplasmic FMR1-interacting protein 1/2	4.22±0.64
Dctn2	Dynactin subunit 2	1.95±0.35
Ddx21	Nucleolar RNA helicase 2	2.14±0.82
Diaph1	Protein diaphanous homolog 1	3.24±0.03
Dnajc13	DnaJ homolog subfamily B member 13	1.56±0.40
Eef1a1/2	Elongation factor 1-alpha 1/2	2.05±0.13
Eif3a	Eukaryotic translation initiation factor 3 subunit A	2.16±0.22
Eif4h	Eukaryotic translation initiation factor 4H	4.05±0.46
Fn1	Fibronectin	2.77±0.32
Ftl1	Ferritin light chain 1	2.40±0.39
Gar1	H/ACA ribonucleoprotein complex subunit 1	1.95±0.44
Gbp2	Interferon-induced guanylate-binding protein 2	3.04±0.31
Hist1h2b	Histone H2B type 1	1.83±0.59
Hnrnpl	Heterogeneous nuclear ribonucleoprotein L	6.31±0.00
Hprt1	Hypoxanthine-guanine phosphoribosyltransferase	1.66±0.18
Myh13	Myosin-13	0.06±0.03
Myh4	Myosin-4	0.09±0.05
Oat	Ornithine aminotransferase, mitochondrial	1.91±0.41
Pa2g4	Proliferation-associated protein 2G4	4.03±0.65
Pdia4	Protein disulfide-isomerase A4	2.37±0.50
Pebp1	Phosphatidylethanolamine-binding protein 1	2.62±0.43
Phgdh	D-3-phosphoglycerate dehydrogenase	2.62±1.11
Ppib	Peptidyl-prolyl cis-trans isomerase B	1.77±0.32
Psma6	Proteasome subunit alpha type-6	1.81±0.24
Psmb1	Proteasome subunit beta type-1	2.02±0.11
Ptges3	Prostaglandin E synthase 3	3.01±0.63
Rbm3	Putative RNA-binding protein 3	1.60±0.08
Rnpep	Aminopeptidase B	2.70±0.78
Rock2	Rho-associated protein kinase 2	6.57±1.45
Rpl13	60S ribosomal protein L13	2.51±0.53
Rpl17	60S ribosomal protein L17	2.04±0.78
Rpl21	60S ribosomal protein L21	2.48±1.10
Rpl23a	60S ribosomal protein L23a	5.09±1.14
Rpl36	60S ribosomal protein L36	1.80±0.55
Rpl36a	60S ribosomal protein L36a	4.03±4.20
Rpl7a	60S ribosomal protein L7a	3.18±0.78
Rpl9	60S ribosomal protein L9	2.28±0.65
Rps25	40S ribosomal protein S25	2.17±1.21
Rps8	40S ribosomal protein S8	2.33±0.67
Snrpd1	Small nuclear ribonucleoprotein Sm D1	2.07±0.63
Tagln	Transgelin	1.90±0.31
Tcp1	T-complex protein 1 subunit alpha	1.94±0.48
Tubb4b	Tubulin beta-4B chain	2.05±0.33
Txnl1	Thioredoxin-like protein 1	2.70±0.59
Ube2n	Ubiquitin-conjugating enzyme E2 N	2.38±0.52

*, expressed as mean ±SEM. Only the Akt2 interaction partners with a fold change greater than 1.5 (i.e., 1.5 fold increase) or less than 0.66 (i.e., 1.5 fold decrease), and with a significant difference of p<0.05, between basal and insulin-treated conditions are considered.

49 proteins showed a significant difference in Akt2 interaction in response to insulin (Table 2); 2 of which were down-regulated, and 47 up-regulated. Among these 49 significantly changed Akt2 interaction partners, 47 were previously unreported in any species.

To help elucidate the potential type of interaction between Akt2 and proteins found in this study, we conducted a search for Akt2 kinase substrates utilizing ScanSite (http://scansite.mit.edu/). The amino acid sequence for each of the 49 significantly enriched Akt2 interaction partners were manually imputed into ScanSite in search of putative Akt kinase motif(s)—R-X-R-X-X-S/T-B—where B is any bulky hydrophobic amino acid residue. The search resulted in a total of 4 potential Akt2 protein substrates (S2 Table). Of the 4proteins containing an Akt phospho motif, elongation factor 1-alpha, eukaryotic translation initiation factor 3 subunit A, fibronectin, and ferritineach showed a significant increase in Akt2 interaction upon insulin stimulation.

**Fig 4:**
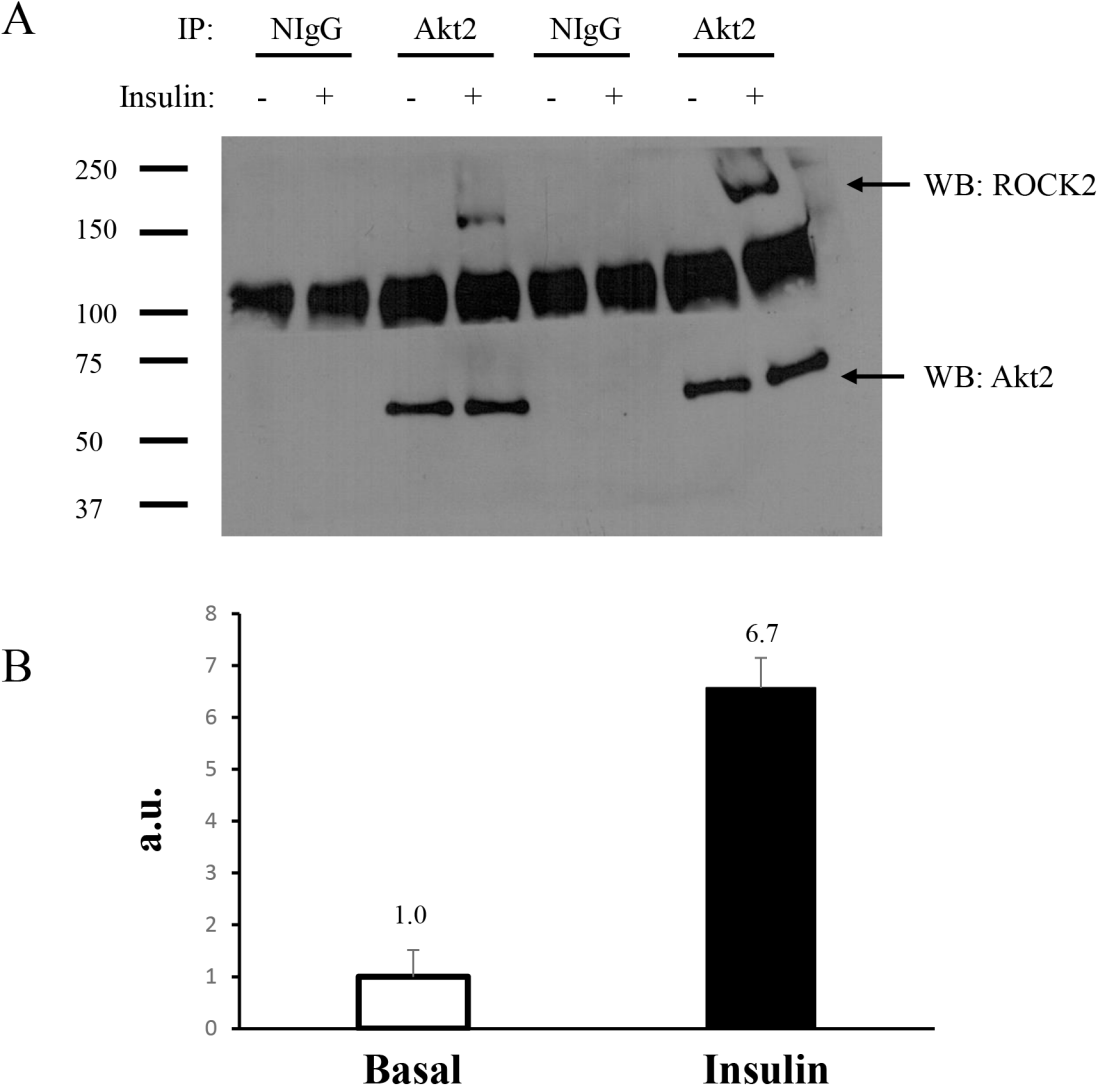
Insulin-stimulated association between Akt2 and ROCK2 in L6 myoblasts. L6 myoblasts were serum-starved for 4h and stimulated with or without insulin (100 nM) for 15 min at 37°C. The cells were lysed and 1 mg of lysates were immunoprecipitated (IP) with normal IgG (NIgG) or Akt2 antibody and western blotted with anti-Akt2 and anti-ROCK2, (A); and relative abundance detected by HPLC-ESI-MS/MS (B).

In the present work, we validated the interaction by immunoprecipitating Akt2 and western blotting for ROCK2. L6 myoblasts were stimulated with or without 100 nM insulin for 15 min and immunoprecipitated with anti-Akt2 antibody followed by immunoblotting with antibodies specific to Akt2 and ROCK2. The results confirmed the insulin-stimulated association of Akt2 and ROCK2 (Fig 4). A band between 100–150kD was displayed for all immunoprecipitates, including NIgG. A monoclonal antibody was chosen as a probe for ROCK2 after a polyclonal antibody (ROCK2 (C-20), sc-1851) was first used. However, the band was still observed. Since the predicted molecular weight of ROCK2 is >160kD, this band is most likely a non-specific band due to the fact that it is below 150kD and present in all immunoprecipitates, including NIgG (a negative control for non-specific binding).

## Discussion

Akt2 plays a critical centralized role in the insulin signaling network. Novel Akt2 interactions may further elucidate insulin signaling and provide insight into abnormal Akt2 protein interaction that contributes to the development of insulin resistance and/or type II diabetes. Skeletal muscle accounts for the a large portion of insulin-mediated postprandial glucose disposal [42]. L6 myoblasts have been shown to be a suitable model for insulin-stimulated GLUT4 translocation as well as glucose uptake, of which Akt2 plays a defining role [31]. The strategy of label free identification offers the ability to detect endogenous proteins interactions, without the use of an overexpressed or tagged “bait” protein, which may lead to a greater probability of non-specific binding and the discovery of false positives [43].

Here, we present 49 proteins that displayed a significant change in Akt2 interaction during insulin stimulation in L6 myoblasts. A list of known, putative Akt2 protein interaction partners was assimilated from multiple databases—Human Protein Reference Database (HPRD), Bio-Grid, PhosphoSite Plus, and IntAct—in order to determine novel Akt2 protein interaction partners from this study. The previously known Akt2 protein interaction partners include a total of 55 proteins from various experimental conditions (in vivo; in vitro; in situ), in multiple cell lines, tissues, and animal models (S1 Table). Multiple techniques for detection of Akt2 partners include, but are not limited to, tandem affinity purification (TAP) MS/MS, Co-IP coupled with western blot, yeast-two hybrid, and proximal ligation assay (PLA). Comparison analysis of previously known interaction partners vs. this study revealed that 47 proteins identified in this study were novel. The identification of a large dataset of novel Akt2 interaction partners indicated multiple pathways that are significantly enriched, such as pathways related to protein synthesis, cell proliferation, cytoskeletal remodeling, and 14-3-3 signaling (Fig 2). Notably, mTOR and p70S6K pathways directly involved in protein synthesis are depicted in S2 and S3 Figs, respectively. Correspondingly, IPA identified the biological function of each of the 49 enriched proteins. The most common biological function categories are shown in Fig 3, which include cell growth and proliferation, cell death and survival, protein synthesis, and cellular movement. Akt2's centralized role in growth and metabolism is highlighted within the enriched pathways as well as most prevalent biological functions. Akt2 is a critical mediator for normal cell growth and proliferation; however, aberrant regulation may lead to other diseases in addition to type 2 diabetes, such as cancer (9). These novel Akt2 interaction partners in L6 myoblasts may help understand the various roles that Akt2 plays in physiological and pathophysiological conditions within muscle as well as other tissue types.

Of the 49 proteins that displayed a significant difference under the influence of insulin stimulation, Rho-associated protein kinase 2 (ROCK2) was the only kinase identified, which displayed a significant increase with Akt2 upon insulin stimulation (Fig 4). Our result is the first report indicating Akt2/ROCK2 interaction. ROCK2 is a serine/threonine kinase, which has been shown to phosphorylate insulin receptor substrate-1 (IRS1), leading to attenuation of insulin signal transduction [44]. Increased activity of ROCK in insulin resistant-induced Wistar rats was associated with increased IRS1 phosphorylation and a decreased active site phosphorylation of Akt [45]. Moreover, increased expression and activity of ROCK2 displayed a concomitant decrease in Akt phosphorylation/activity in diabetic bone marrow endothelial cells [46]. Overall, ROCK2 is suggested to play a negative role in insulin signaling due to its negative feedback on IRS1. Insulin-stimulated Akt2 association with other kinases, such as mTOR, S6K1, and GSK3, also lead to subsequent IRS1 site-specific hyper-serine/threonine phosphorylation [2]. ROCK2 does not contain a putative Akt2 phospho motif (S2 Table), neither does Akt2 contain a putative ROCK2 phospho motif (R/KXS/T; R/KXXS/T), which suggests the interaction of Akt2-ROCK2 is not potentially mediated by phosphorylation. Instead, Akt2-ROCK2 interaction may be indirect, mediated through the interaction of Cdc42, a known ROCK2 interaction partner [47] that also was identified in this study. Nonetheless, ROCK2 plays a role in actin cytoskeletal rearrangement [47], which may be important for GLUT4 translocation and subsequent glucose uptake.

**Fig 5:**
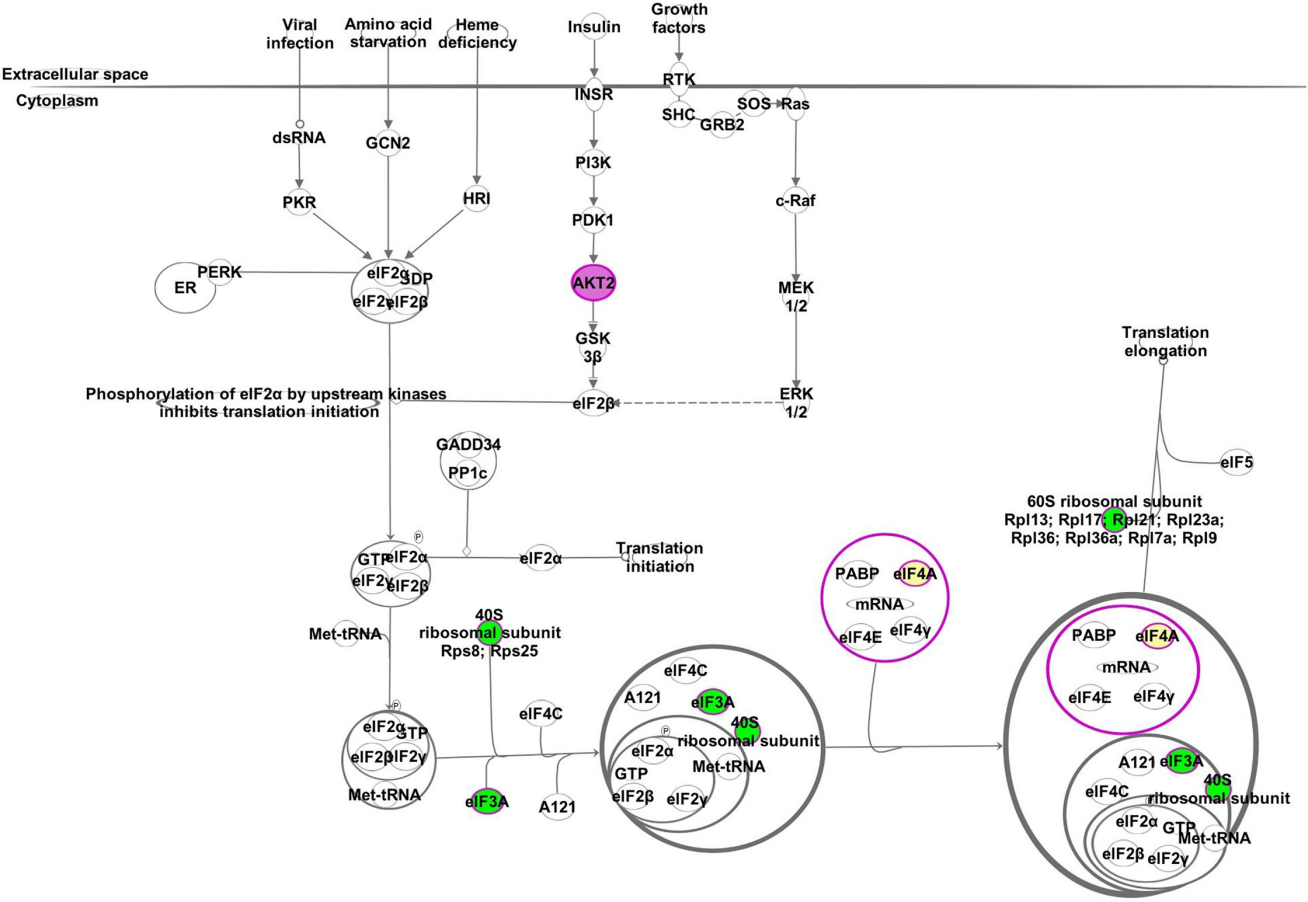
Significantly enriched canonical pathway, EIF2, for the Akt2 interaction partners identified in this study. Pathway analysis was revealed by proteomics and Ingenuity Pathways Analysis. Akt2 is highlighted in purple. Proteins with increased interaction to Akt2 after the insulin treatment are highlighted in green, proteins with decreased interaction to Akt2 after the insulin treatment are highlighted in red, and identified interaction partners with no differences in their interaction to Akt2 under the basal and insulin stimulated conditions are highlighted in yellow. Proteins without color are identified in the network IPA database, but were not identified in this study.

Multiple proteins identified as Akt2 interaction partners form a portion of the eukaryotic initiation factor 2 (eIF2) heterotrimer complex, as well as multiple additional proteins that play a role in protein synthesis (Fig 5), including 40S ribosomal proteins (Rps8 and Rps25) and 60S ribosomal proteins (Rpl7a, Rpl9, Rpl13, Rpl17, Rpl21, Rpl23A, Rpl36, and Rpl36a). The eIF2 complex initiates translation through the binding of Met-tRNA to the 40S ribosomal subunit, which is activated in a GTP-dependent manner [48]. The pre-initiation complex (PIC) is then stimulated by initiation factors, such as eIF3, which then are able to bind mRNA. The PIC complex then binds eIF4 to form a 48S/mRNA complex that subsequently binds eIF5, which hydrolyzes GTP to GDP [49]. Once GTP hydrolysis occurs, the 60S ribosomal subunit is recruited to carry out translation elongation (i.e., protein synthesis). The eIF2 complex is regulated by multiple serine kinases, such as protein kinase double-stranded RNA-dependent (PKR) (Donnelly et al., 2013). Our results indicate that Akt2 potentially regulates multiple regulatory proteins (eIF3, eIF4, eIF5, 40S, 60S) involved in translation initiation as well as translation elongation (Fig 5). Additionally, Akt2 significantly increased binding to eIF3, 40S, and 60S proteins upon insulin stimulation.

It is well established that activated tyrosine kinase receptors, such as insulin receptor (IR)/insulin-like growth factor-1 receptor (IGF-IR), stimulate protein synthesis through the PI-3K pathway, notably within insulin responsive tissues such as skeletal muscle [50]. Akt2, which plays a centralized role in the PI-3K pathway stimulates mTOR activity, which then stimulates p70S6K activityresulting in subsequent protein synthesis (S2 and S3 Figs). Akt2's role downstream of mTOR directly interacting with protein synthesis constellations will need to be further investigated. However, in contrast to these findings, upon IGF-I stimulation in embryonic rat cardiomyocytes, Akt2 was shown to dissociate from elongation factor 2 (EF2) but was stabilized with PI-3K inhibition [51]. This, taken with the results from this study, suggests that Akt2 interacts with and potentially regulates protein synthesis complexes in an isoform- and insulin-dependent manner.

The present project analyzes proteins isolated from insulin sensitive L6 myoblasts under basal and insulin-stimulated conditions utilizing state-of-the-art HPLC-ESI-MS/MS to assess endogenous interaction partners of Akt2, a keystone mediator in the insulin signaling cascade. We identified 49 Akt2 interaction partners displayed a significant change in Akt2 interaction following insulin stimulation, including proteins that play a role in protein synthesis, carbohydrate metabolism, muscle contraction and cytoskeleton rearrangement, protein degradation, and protein folding. These results identified multiple novel endogenous insulin-stimulated Akt2 interaction partners, providing targets to investigate Akt2 interaction partners in animal models and humans as well as new insights into insulin signaling.

## Supporting Information

S1 Fig One dimensional-SDS-PAGE gels for NIgG control and Akt2 co-immunoprecipitation. Please note that preclearing was used not only to reduce the unspecific background, but also to identify non-specific binder.
(TIF)

S2 Fig A significantly enriched canonical pathway, mTOR, for the Akt2 interaction partners identified in this study. Pathway analysis was revealed by proteomics data and Ingenuity Pathway Analysis. Akt2 is highlighted in purple. Proteins with increased Akt2 insulin-stimulated interaction are highlighted in green, proteins with decreased insulin-stimulated interaction to Akt2 are highlighted in red, and identified interaction partners with no change in their interaction to Akt2 under the basal and insulin treatment conditions are highlighted in yellow. Proteins without color were not identified in this study but found in the network in the IPA database.
(TIF)

S3 Fig A significantly enriched canonical pathway, p70S6K, for the Akt2 interaction partners identified in this study. Pathway analysis was revealed by proteomics data and Ingenuity Pathway Analysis. Akt2 is highlighted in purple. Proteins with increased Akt2 insulin-stimulated interaction are highlighted in green, proteins with decreased insulin-stimulated interaction to Akt2 are highlighted in red, and identified interaction partners with no change in their interaction to Akt2 under the basal and insulin treatment conditions are highlighted in yellow. Proteins without color were not identified in this study but found in the network in the IPA database.
(TIF)

S1 Table Previously reported Akt2 interaction partners. (XLSX)

S2 Table Four of the 49 significantly enriched proteins that contain an Akt phospho motif. Data was compiled using ScanSite.
(XLSX)
