# Supplementary figures and images for: Novel Endogenous, Insulin-Stimulated Akt2 Protein Interaction Partners in L6 Myoblasts
Source: PLoS One. 2015 Oct 14;10(10):e0140255. doi: 10.1371/journal.pone.0140255 (PMC4605787; doi:10.1371/journal.pone.0140255)

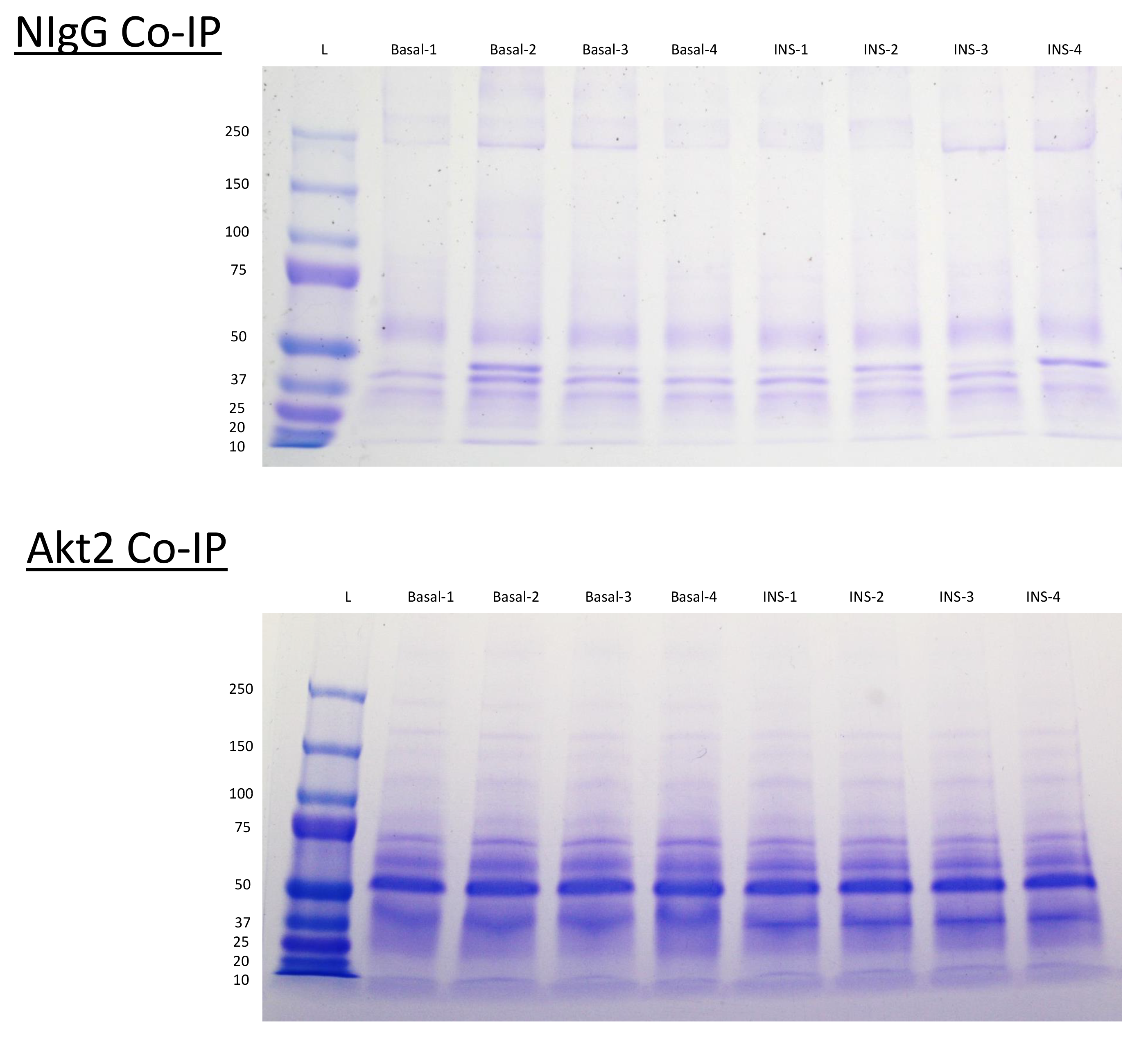

Supplement: S1 Fig — Please note that preclearing was used not only to reduce the unspecific background, but also to identify non-specific binder. (TIF) [file pone.0140255.s001.tif]

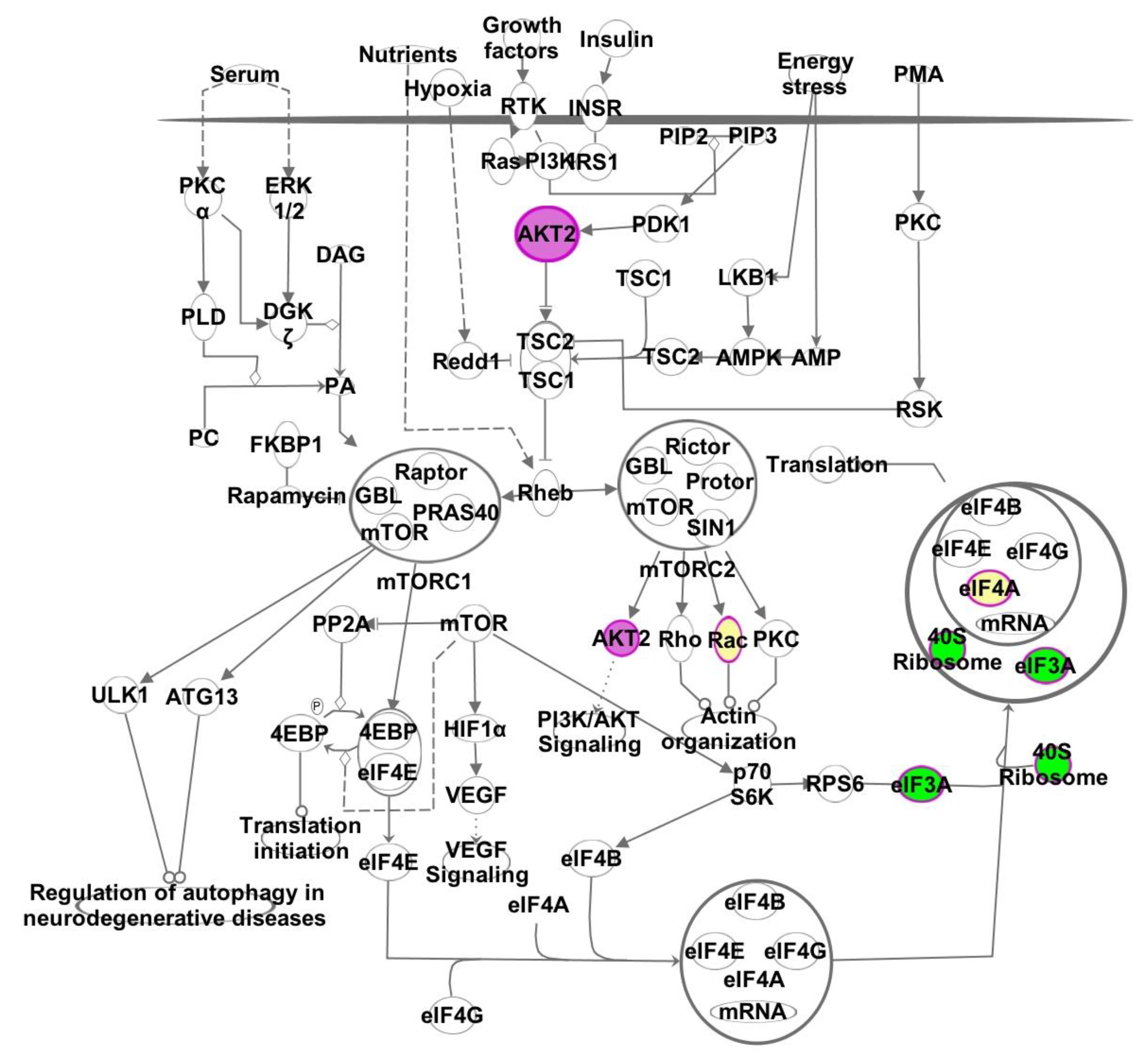

Supplement: S2 Fig — Pathway analysis was revealed by proteomics data and Ingenuity Pathway Analysis. Akt2 is highlighted in purple. Proteins with increased Akt2 insulin-stimulated interaction are highlighted in green, proteins with decreased insulin-stimulated interaction to Akt2 are highlighted in red, and identified interaction partners with no change in their interaction to Akt2 under the basal and insulin treatment conditions are highlighted in yellow. Proteins without color were not identified in this study but found in the network in the IPA database. (TIF) [file pone.0140255.s002.tif]

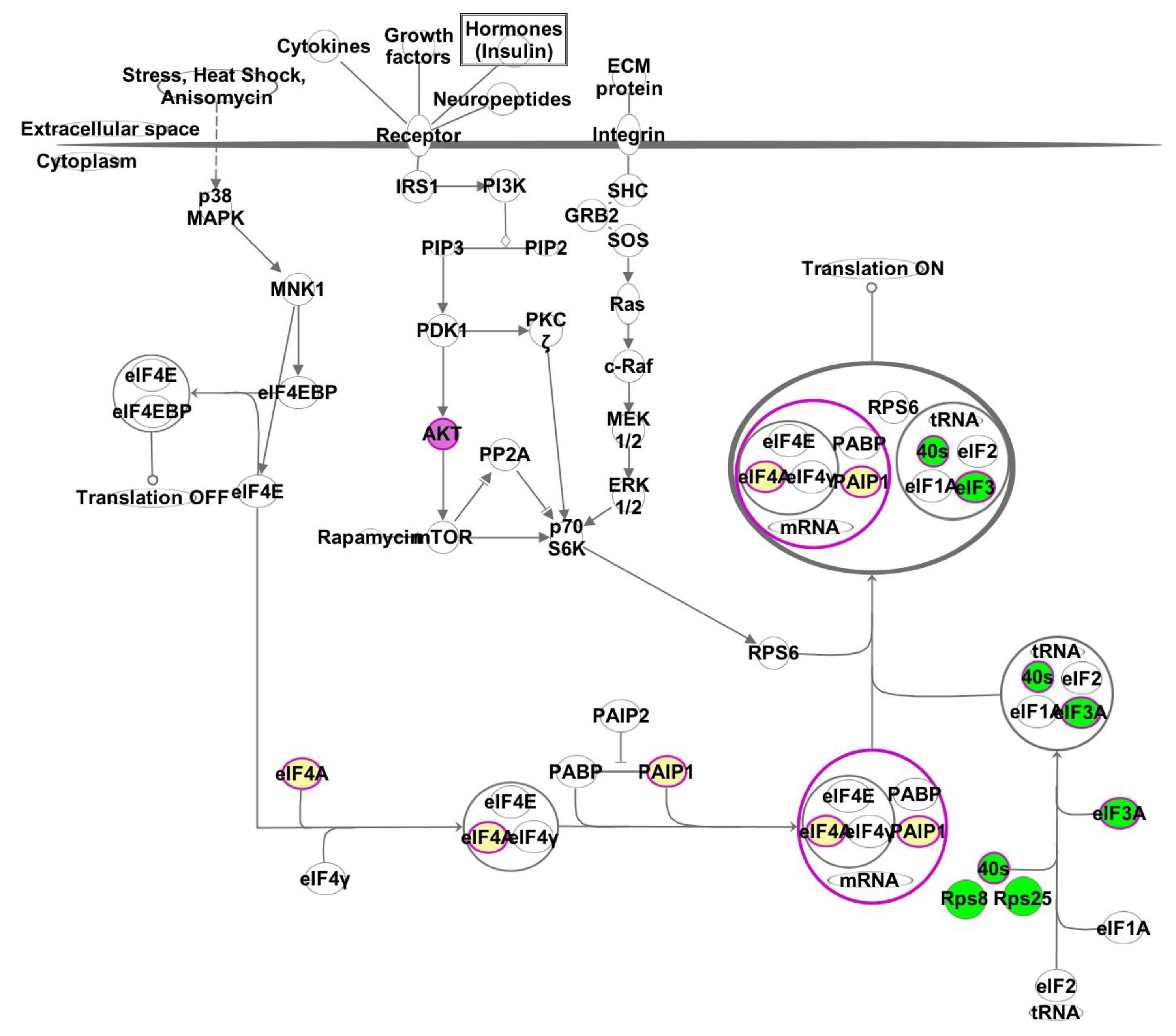

Supplement: S3 Fig — Pathway analysis was revealed by proteomics data and Ingenuity Pathway Analysis. Akt2 is highlighted in purple. Proteins with increased Akt2 insulin-stimulated interaction are highlighted in green, proteins with decreased insulin-stimulated interaction to Akt2 are highlighted in red, and identified interaction partners with no change in their interaction to Akt2 under the basal and insulin treatment conditions are highlighted in yellow. Proteins without color were not identified in this study but found in the network in the IPA database. (TIF) [file pone.0140255.s003.tif]
